# Construction of developmentally inspired periosteum-like tissue for bone regeneration

**DOI:** 10.1038/s41413-021-00166-w

**Published:** 2022-01-03

**Authors:** Kai Dai, Shunshu Deng, Yuanman Yu, Fuwei Zhu, Jing Wang, Changsheng Liu

**Affiliations:** 1 State Key Laboratory of Bioreactor Engineering, East China University of Science and Technology, Shanghai, P. R. China; 2 Engineering Research Center for Biomedical Materials of Ministry of Education, East China University of Science and Technology, Shanghai, P. R. China; 3 Key Laboratory for Ultrafine Materials of Ministry of Education, East China University of Science and Technology, Shanghai, P. R. China; 4 Frontiers Science Center for Materiobiology and Dynamic Chemistry, East China University of Science and Technology, Shanghai, P. R. China

**Keywords:** Bone, Bone quality and biomechanics

## Abstract

The periosteum, a highly vascularized thin tissue, has excellent osteogenic and bone regenerative abilities. The generation of periosteum-mimicking tissue has become a novel strategy for bone defect repair and regeneration, especially in critical-sized bone defects caused by trauma and bone tumor resection. Here, we utilized a bone morphogenetic protein-2 (BMP-2)-loaded scaffold to create periosteum-like tissue (PT) in vivo, mimicking the mesenchymal condensation during native long bone development. We found that BMP-2-induced endochondral ossification plays an indispensable role in the construction of PTs. Moreover, we confirmed that BMP-2-induced PTs exhibit a similar architecture to the periosteum and harbor abundant functional periosteum-like tissue-derived cells (PTDCs), blood vessels, and osteochondral progenitor cells. Interestingly, we found that the addition of chondroitin sulfate (CS), an essential component of the extracellular matrix (ECM), could further increase the abundance and enhance the function of recruited PTDCs from the PTs and finally increase the regenerative capacity of the PTs in autologous transplantation assays, even in old mice. This novel biomimetic strategy for generating PT through in vivo endochondral ossification deserves further clinical translation.

## Introduction

The periosteum serves as an attachment surface for skeletal muscles and ligaments and covers almost the entire bone surface.^1,2^ Moreover, the periosteum harbors multiple cell types, including osteogenic cells and fibroblastic cells, blood vessels, and nerve endings, which play a central role in bone fracture and defect repair.^3–7^ As a thin membrane covers the bone, the periosteum also consists of multiple extracellular matrices (ECMs), such as collagens and glycosaminoglycans.^8,9^ Periosteum-derived cells (PDCs) or periosteum-derived multipotent mesenchymal stromal cells (MSCs), as a crucial component of the periosteum, are heterogeneous and may contain different subpopulations of multipotent stem cells.^10,11^ PDCs are deeply involved in the generation of bone, cartilage, and hematopoietic marrow.^5,7^ A recent study identified a multipotent and self-renewing type of periosteal stem cell that emerges in the periosteum.^12^ Other studies have indicated that leptin receptor-positive (LepR^+^) cells, which are also strongly positive for CD140α (PDGFR-α) and CD105, are the major subpopulations of MSCs that form bone and adipocytes in adult bone marrow.^13–15^ LepR^+^ cells found in the periosteum may serve as a subpopulation of PDCs that are essential for bone formation and regeneration.^10^ Functional blood vessels, such as type H (strongly positive for CD31 and endomucin; CD31^hi^EMCN^hi^) vessels, are another vital component in the periosteum that couple angiogenesis and osteogenesis during bone development and repair processes.^10,16,17^ Therefore, the periosteum exhibits an impressive bone regenerative capacity and can be a potential cellular source for bone regenerative applications.^18,19^

The design and fabrication of periosteum-like tissue (PT) could be an effective strategy for bone repair and reconstruction inspired by the strong bone regenerative capacity of the periosteum.^1,20–24^ Clinically, the induced membrane (IM) technique is a mature therapy for trauma-, bone tumor-, and osteomyelitis-triggered large bone defects that activates the immune system of individuals to induce vascularized granulation tissue.^25–27^ This technology utilizes an implanted cement spacer, commonly polymethylmethacrylate, to induce PT at the first stage and involves implantation of the autologous bone graft with bone marrow aspirate into the defect area after the removal of the cement spacer at the second stage, but it is still limited by the incubation time, which is typically over 6 weeks, and the complex surgical procedures.^23,28,29^ Other studies have generated PTs utilizing synthetic membranes with or without exogenous cells, which have a limited regenerative capacity and require intensive manipulation due to the incorporation of exogenous cells.^1,20,21,30,31^ A previous study confirmed that human periosteal cell-loaded scaffolds could induce ectopic bone formation in vivo.^32^ A recent study reported that bone morphogenetic protein-2 (BMP-2)-induced heterotopic ossification (HO) originates from abundant recruited multipotent stromal cells coupled with type H vessels.^33^ Inspired by this interesting discovery, we hypothesized that we could utilize BMP-2 to directly and effectively activate the residual regenerative capacity of individuals to construct multipotent stromal cell-rich and vascularized PT in vivo.

In this study, we sought to construct PT in vivo using a material strategy involving BMP-2-initiated endochondral ossification. Abundant periosteum-like tissue-derived cells (PTDCs) can be recruited spontaneously, along with functional blood vessels invading the PT. We attempted to further regulate the function of PTs and found that chondroitin sulfate (CS), as an enhancer, could increase the abundance and promote the osteochondral differentiation and recruitment capacity of recruited PTDCs from PTs. Autologous transplantation assays also confirmed that the PTs induced by the BMP-2/CS-loaded scaffold resulted in enhanced osteogenesis and osseointegration related to the BMP-2-induced compartment, even in old mice. This strategy for creating in vivo PT could be further applied to repair complex craniofacial defects, especially for elderly patients.

## Results

### Construction of PTs in vivo

**Fig. 1:**
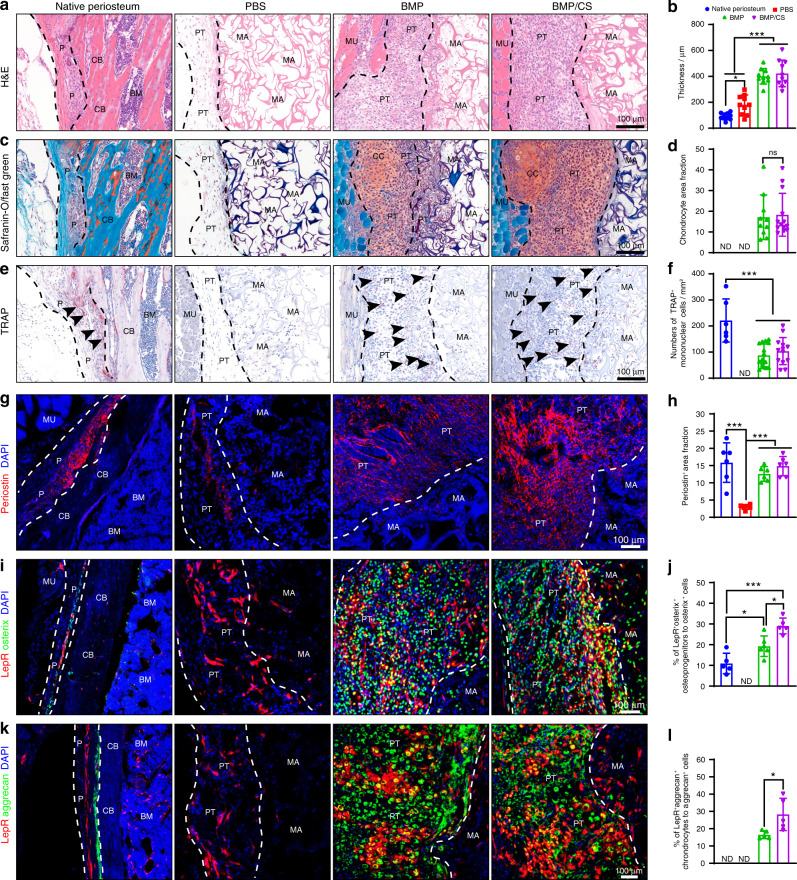
PTs possess a similar architecture to the native periosteum. **a**–**f** Histological staining and quantitative analysis of native femur periosteum and induced tissues from the *Lepr*-cre; tdTomato mice for H&E (**a**, **b**), Safranin O/Fast Green (**c**, **d**), and tartrate-resistant acid phosphatase (TRAP) (**e**–**f**) staining (*n* = 6–10) in the PBS, BMP, and BMP/CS groups. **g**–**l** Immunofluorescence staining and quantitative analysis of periostin (red; **g**, **h**), LepR^+^ cell-derived osteoprogenitors (LepR^+^Osterix^+^, yellow; **i**, **j**), and LepR^+^ cell-derived chondrocytes (LepR^+^Aggrecan^+^, yellow; **k**, **l**) in the native femur periosteum, PBS, BMP, and BMP/CS groups in the *Lepr*-cre; tdTomato mice (*n* = 5–13). Periostin- and LepR-positive cells are red, Osterix- or Aggrecan-positive cells are green, and DAPI is blue (nucleus). Periosteum (P), periosteum-like tissue (PT), cortical bone (CB), chondrocyte (CC), material (MA), muscle (MU), not detected (ND). The black dashed line indicates the native periosteum and induced tissues (**a**, **c**, **e**). The black arrowhead indicates TRAP-positive cells (**e**). The white dashed line indicates the border of the periosteum and cortical bone or PT and material (**g**, **i**, **k**). Scale bar, 100 μm. Data are presented as the mean ± SD. *P < 0.05, **P < 0.01, ***P < 0.001, one-way ANOVA followed by Tukey’s post hoc tests or two-tailed Student’s *t* test

Previous studies have shown that BMP-2 induces ectopic bone formation via endochondral ossification.^15,34^ CS has been reported to regulate the osteogenic capacity of BMP-2 in bone defect repair.^35,36^ LepR has been utilized as an effective marker for enriching PDCs.^10,14^ To confirm the induction of PTs via a biomaterial strategy, we subcutaneously implanted all three types of scaffolds (PBS-loaded gelatin scaffold, BMP-loaded gelatin scaffold, and BMP/CS-loaded gelatin scaffold) into *Lepr*-cre; tdTomato mice (Supplementary Fig. 1). The induced PTs were proven to possess similar architecture to native periosteum and abundant functional cells, such as LepR^+^ progenitor cells, osteoprogenitors, and chondrocytes (Fig. 1). We found that the implanted scaffolds were surrounded by granulation tissue and that the granulation tissues from the BMP and BMP/CS groups were much thicker than the native periosteum and granulation tissues from the PBS group, whereas the thickness of the granulation tissue between the BMP and BMP/CS groups showed no significant difference (Fig. 1a, b). The PTs from the BMP and BMP/CS groups exhibited a typical compact fibrous architecture that was similar to that of the native femur periosteum.

Safranin O and TRAP staining further illustrated that mesenchymal cells condensed and formed PT at the early stage, typically 1 week after implantation (Fig. 1c–f). Some chondrocytes were embedded in the PTs from the BMP and BMP/CS groups (Fig. 1c). TRAP-positive cells were also identified in the PTs from the BMP and BMP/CS groups, which may contribute to the degradation of gelatin scaffolds, recruitment of PDCs, and invasion of blood vessels (Fig. 1e). Although all three types of scaffolds could induce granulation tissue, which had a similar architecture to the periosteum, we found abundant periostin, LepR^+^ progenitor cells, osteoprogenitors, and chondrocytes lodged in the PTs in the BMP and BMP/CS groups, whereas little periostin and no osteoprogenitors or chondrocytes were found in granulation tissue of the PBS group (Fig. 1c–i). We still found that some LepR^+^ progenitor cells migrated into the granulation tissue of the PBS group, which could be explained by the implanted area being injured and the LepR^+^ progenitor cells preferentially migrating into the injury area spontaneously for tissue regeneration. These results demonstrated that BMP-2 was indispensable for inducing PTs and confirmed that both the BMP- and BMP/CS-loaded scaffolds could spontaneously generate PTs in vivo. LepR^+^ progenitor cells in PTs were also confirmed to show osteogenic and chondrogenic differentiation.

### PTs harbor abundant functional PTDCs

**Fig. 2:**
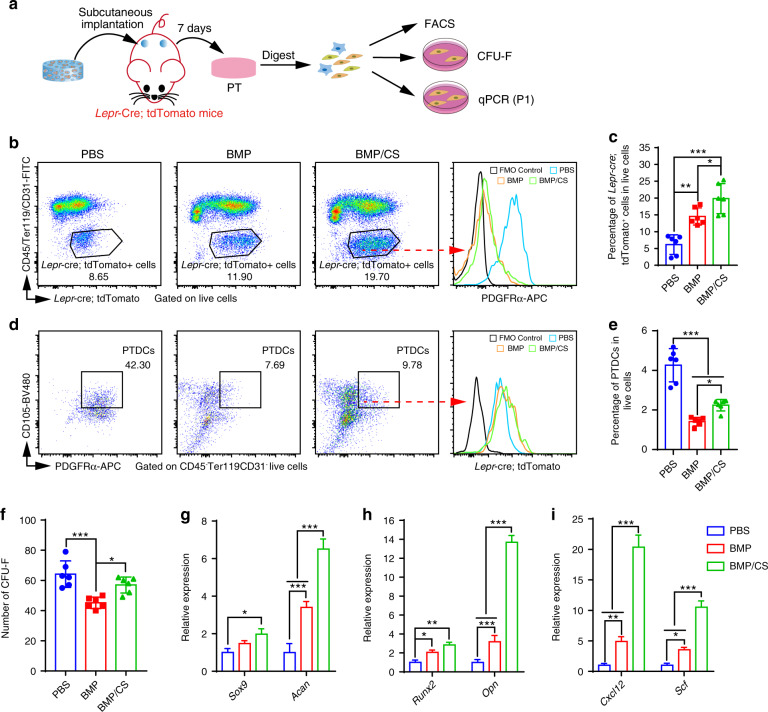
Functional PTDCs are lodged in PTs. **a** Scheme of the experiments for evaluating the function of PTDCs in PTs from the PBS, BMP, and BMP/CS groups. **b**–**e** Representative flow cytometric profiles and quantitative analysis of *Lepr*-cre; tdTomato^+^ cells (**b**, **d**) and PTDCs (CD45^−^Ter119^−^CD31^−^CD140a ^+^CD105 ^+^; **c**, **e**) from the PBS, BMP, and BMP/CS groups 1 week after subcutaneous implantation in *Lepr*-cre; tdTomato mice (*n* = 6). The *Lepr*-cre; tdTomato^+^ cells and CD140a ^+^CD105 ^+^PTDCs were gated on CD45^−^Ter119^−^CD31^−^ live cells with the elimination of debris, dead cells, and doublets/cell aggregates. Data from individual animals are shown as the mean ± SD. *P < 0.05, **P < 0.01, ***P < 0.001, one-way ANOVA followed by Tukey’s post hoc tests. **f** Number of colony-forming unit fibroblasts (CFU-Fs) per 1 × 10^4^ enzymatically dissociated cells from the PBS, BMP and BMP/CS groups (*n* = 6). Data from individual animals are shown as the mean ± SD. *P < 0.05, **P < 0.01, ***P < 0.001, one-way ANOVA followed by Tukey’s post hoc tests. **g**–**i** qPCR analysis of the transcript levels of genes associated with chondrogenesis (**g**), osteogenesis (**h**), and stem cell maintenance (**i**) in PTDCs from PTs in the PBS, BMP, and BMP/CS groups (*n* = 3). Total RNA was extracted from first-passage PTDCs. Data from individual animals are shown as the mean ± SD. *P < 0.05, **P < 0.01, ***P < 0.001, two-way ANOVA followed by Bonferroni’s post hoc tests

To further verify the abundance and function of PTDCs in the PTs explanted from the *Lepr*-cre; tdTomato mice among the PBS, BMP, and BMP/CS groups, we conducted flow cytometry, CFU-F assays, and qPCR assays (Fig. 2a). A previous study reported that *Lepr*-cre; tdTdtomato^+^ cells could label PDCs derived from native periosteum.^10^ However, in this study, we found that *Lepr*-cre; tdTdtomato^+^ cells could label only a portion of PTDCs in the BMP and BMP/CS groups but labeled nearly 100% of PTDCs in the PBS group (Fig. 2b and Supplementary Fig. 2). This phenomenon may be due to the fact that a large portion of PTDCs in the BMP and BMP/CS groups differentiated into osteochondral progenitor cells with the activation of BMP-2 and CS and did not express PTDC markers, such as PDGFRα. Given this fact, *Lepr*-cre; tdTdtomato^+^ cells could label the total PTDCs in the PTs, which consist of differentiated and undifferentiated PTDC subpopulations. The flow cytometric data confirmed that the fraction of LepR^+^ cells in the BMP (14.53% ± 2.57%) and BMP/CS (19.83% ± 4.49%) groups was much higher than that in the PBS group (6.12% ± 2.92%; Fig. 2c). To further evaluate the abundance of undifferentiated PTDCs in the PBS, BMP, and BMP/CS groups, we defined the phenotype of undifferentiated PTDCs derived from PTs as CD45^−^Ter119^−^CD31^−^CD140a ^+^ CD105 ^+^ and positive for LepR-tdTomato (Fig. 2d and Supplementary Fig. 2). We found that the PTs in the BMP/CS group harbored a higher fraction of PTDCs than those in the BMP group (2.24% ± 0.29% vs. 1.41% ± 0.21%; Fig. 2e). However, the fraction of PTDCs in the PBS group had the highest fraction of PTDCs among all three groups, which indicated that the migrated PTDCs in the PBS group remained undifferentiated and did not undergo osteogenic and chondrogenic processes (Fig. 2e).

The number of CFU-F colonies in the PBS group was the highest among all three groups (64.17% ± 8.78%) since the majority of LepR^+^ cells in the PBS group were undifferentiated PTDCs (Fig. 2f). The number of CFU-F colonies in the BMP/CS group was still higher than that in the BMP group, which was consistent with the flow cytometric results (57.00 ± 5.30 vs. 45.17 ± 3.77; Fig. 2f). These data confirmed the high fraction and strong self-renewal capacity of PTDCs from the in vivo-produced PTs. Moreover, the PTDCs from the BMP/CS group possessed a stronger self-renewal capacity than the PTDCs from the BMP group.

We then conducted qPCR analyses to evaluate the functional gene expression of PTDCs derived from PTs in the PBS, BMP, and BMP/CS groups (Fig. 2g–i). For chondrogenic genes, such as *Sox9* and *Acan*, we found that the PTDCs from the BMP/CS group expressed similar levels of the *Sox9* gene as those from the BMP group, but the expression level of the *Acan* gene increased 1.91-fold compared to that of the PTDCs from the BMP group (Fig. 2g). The levels of the *Sox9* and *Acan* genes in PTDCs from the BMP/CS group were much higher than those in PTDCs from the PBS group, with increases of 1.98- and 6.51-fold, respectively (Fig. 2g). For osteogenic genes, such as *Runx2* and *OPN*, we found that the expression levels in the PTDCs from the BMP and BMP/CS groups were much higher than those in the PDCs from the PBS group. Moreover, the PTDCs from the BMP/CS group expressed the highest levels of osteogenic genes among the three groups. (Fig. 2h).

Moreover, we found that the expression of classic stem cell maintenance-associated genes, such as *CXCL12* and *SCF*, in the PTDCs from the BMP/CS group was much higher than that in the PDCs from the PBS (20.36- and 10.54-fold increases) and BMP (4.14- and 2.97-fold increases) groups (Fig. 2i). All the quantitative data above confirmed that PTs in both the BMP and BMP/CS groups harbored abundant functional PTDCs and that CS could further increase the fraction of PTDCs in newly generated PTs. The high expression level of chondrogenic, osteogenic, and stem cell maintenance-associated genes in the PTDCs derived from the PTs in the BMP/CS group may indicate that these PTs exhibited an enhanced capacity for bone defect repair relative to the PTs from the BMP group.

**Fig. 3:**
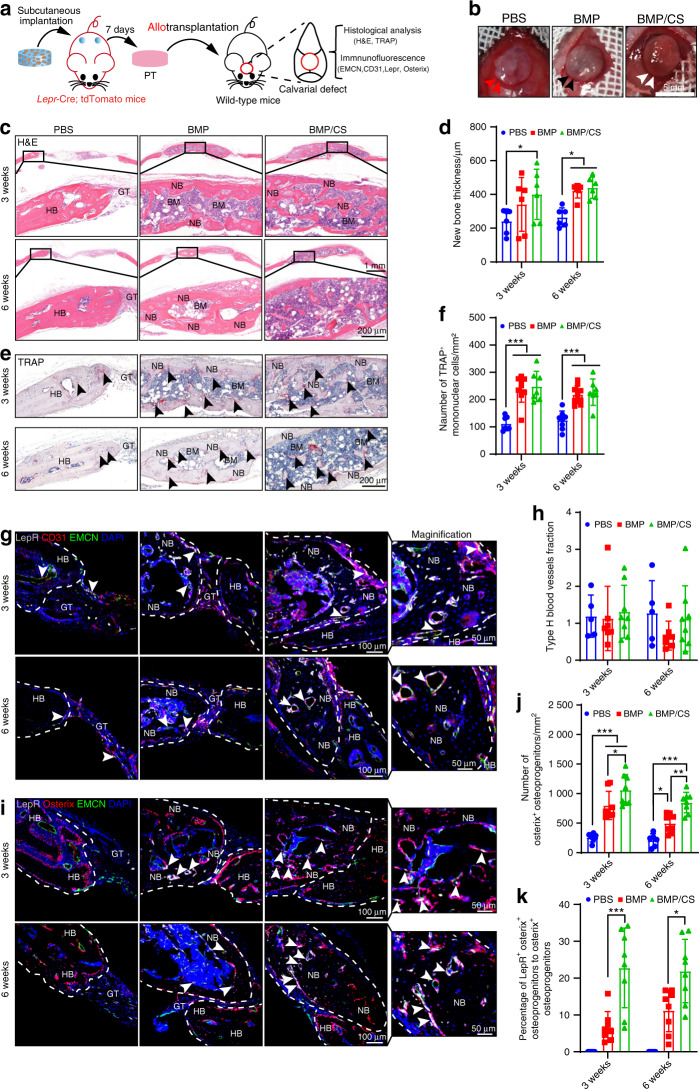
Functional PTDCs lodged in PTs participate in osteogenesis and osseointegration during allogenic transplantation. **a** Scheme of the experiments for allogenic transplantation with the PTs from the PBS, BMP, and BMP/CS groups. Freeze-dried PBS-, BMP-, or BMP/CS-loaded scaffolds were subcutaneously implanted in *Lepr*-cre; tdTomato mice for 1 week (*n* = 6). Then, the obtained PTs were engraved for transplantation. **b** Surgery for allogenic transplantation for critical-sized calvarial defect repair in wild-type mice. Images were taken at the initial surgical procedure. The red arrowhead indicates animals transplanted with allogenic PT from the PBS group, the black arrowhead indicates animals transplanted with allogenic PT from the BMP group, and the white arrowhead indicates animals transplanted with allogenic PT from the BMP/CS group. **c**–**f** Representative images and quantitative analysis of H&E (**c**, **d**) and TRAP (**e**, **f**) staining of cranial samples from the PBS, BMP, and BMP/CS groups at weeks 3 and 6 after allogenic transplantation in wild-type mice. **g**–**k** Representative images and quantitative analysis of immunofluorescence staining of type H vessels (EMCN ^+^ CD31 ^+^, yellow; **g**, **h**), Osterix^+^ osteoprogenitors (red; **i**, **j**) and *Lepr*-cre; tdTomato donor-derived osteoprogenitors (**i**, **k**) in the PBS, BMP, and BMP/CS groups at weeks 3 and 6 after allogenic transplantation in young mice. Granulation tissue GT, bone marrow BM, new bone NB, host bone HB. The black arrowhead indicates TRAP-positive cells (**e**). *Lepr*-cre; tdTomato-positive cells are white, EMCN-positive cells are green, CD31- and Osterix-positive cells are red, and DAPI is blue (nucleus). The white dashed line indicates the host bone or new bone area (**g**, **i**). The white arrowhead indicates type H blood vessels (**g**) and LepR^+^Osterix^+^ osteoprogenitors (**i**). Scale bar (top: low magnification, 1 mm; bottom: high magnification, 200 μm; **c**, **e**) and (left: low magnification, 100 μm; right: high magnification, 50 μm; **g**, **i**). Biological replicates are 5–8. Data are presented as the mean ± SD. *P < 0.05, **P < 0.01, ***P < 0.001, two-way ANOVA followed by Bonferroni’s post hoc tests

To further evaluate the fate of PTDCs derived from PTs during bone reconstruction, we traced the transplanted PTDCs via lineage-tracing technology and in vivo imaging in allogenic transplantation assays. We utilized *Lepr*-Cre; tdTomato mice to label the PTDCs in the PTs from the PBS, BMP, and BMP/CS groups and transplanted newly generated PTs onto the critical-sized calvarial defect area (Fig. 3a, b). Histological analysis confirmed that transplanted PTs in the BMP and BMP/CS groups could differentiate into bone tissue as early as 3 weeks after transplantation (Fig. 3c, e). The thickness of new bone in the BMP and BMP/CS groups was higher than that in the PBS group but was not significantly different between the BMP and BMP/CS groups (Fig. 3d). Moreover, newly generated bone from both the BMP and BMP/CS groups underwent active bone remodeling at all indicated time points, and the number of TRAP ^+^ mononuclear cells was much higher in the BMP and BMP/CS groups than in the PBS groups (Fig. 3e, f).

Further immunofluorescence staining confirmed that the transplanted LepR^+^ PTDCs derived from PTs could achieve long-term survival after transplantation accompanied by abundant blood vessels (Fig. 3g, h). Moreover, we observed that the newly generated bone area in both the BMP/CS group and the BMP group harbored many LepR^+^ PTDC-derived cells and Osterix^+^ osteoprogenitors (Fig. 3i, j–k). Specifically, the number of Osterix^+^ osteoprogenitors and the LepR^+^Osterix^+^ osteoprogenitor fraction in the BMP/CS group were significantly higher than those in the PBS and BMP groups, and no LepR^+^Osterix^+^ osteoprogenitors were found in the PBS group. In vivo imaging data also confirmed that the LepR^+^ PDTCs derived from the transplanted PTs in the BMP and BMP/CS groups could survive and participate in bone reconstruction at week 6 after transplantation (Supplementary Fig. 3). The allotransplantation assay confirmed that the LepR^+^ PTDCs derived from the transplanted PTs could steadily differentiate into Osterix^+^ cells and participate in new bone generation after transplantation.

### PTs harbor abundant functional blood vessels and osteochondral progenitor cells

**Fig. 4:**
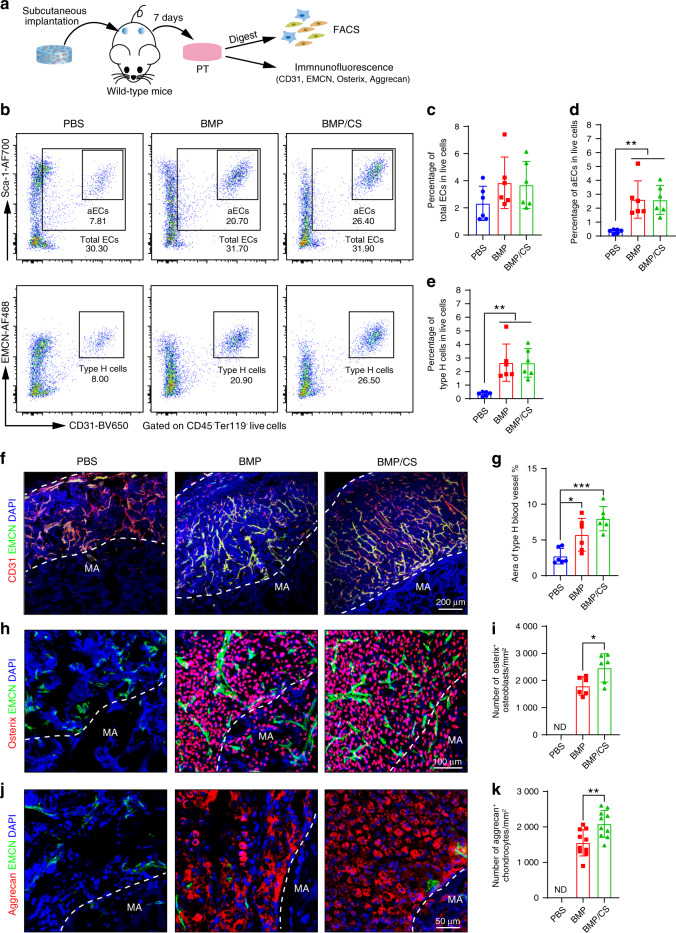
Evaluation of functional blood vessels and osteochondral progenitor cells in PTs. **a** Scheme of the experiments for evaluating the abundance of blood vessels and osteochondral progenitor cells in PTs from the PBS, BMP, and BMP/CS groups. **b** Representative flow cytometric profiles of total endothelial cells (total ECs), arteriolar ECs (aECs) and type H cells of the PBS, BMP, and BMP/CS groups 1 week after subcutaneous implantation in wild-type mice (*n* = 6). **c**–**e** Quantitative flow cytometric analysis of total ECs (Ter119^−^CD45^−^CD31 ^+^ Sca-1^+^, **c**), aECs (Ter119^-^CD45^-^CD31^hi^Sca-1^hi^, **d**) and type H blood cells (Ter119^-^CD45^-^CD31 ^+^ EMCN ^+^, **e**) in the BMP and BMP/CS groups 1 week after subcutaneous implantation in young wild-type mice. Data are from individual animals and are shown as the mean ± SD. **P < 0.01, one-way ANOVA followed by Tukey’s post hoc tests. **f**–**k** Immunofluorescence staining and quantitative analysis of type H blood vessels (EMCN ^+^ CD31 ^+^, yellow; **f**, **g**), Osterix^+^ osteoprogenitors (red; **h**, **i**), and Aggrecan^+^ chondrocytes (red, **j**, **k**) in the PBS, BMP, and BMP/CS groups 1 week after subcutaneous implantation in wild-type mice (*n* = 6–8). CD31-positive cells, Osterix-positive cells, and Aggrecan-positive cells are red, EMCN-positive cells are green, and DAPI is blue (nucleus). Materials (MA). The white dashed line indicates the border of the material area and PT area. Data are from individual animals and are shown as the mean ± SD. *P < 0.05, **P < 0.01, ***P < 0.001, one-way ANOVA followed by Tukey’s post hoc tests. Scale bar, 200 μm (**f**), 100 μm (**h**), and 50 μm (**j**)

The periosteum has been proven to be a fibrous tissue that harbors abundant blood vessels and skeletal stem and progenitor cells.^5–7^ To explore the occurrence of vasculature, as well as skeletal stem and progenitor cells, in the PTs from the PBS, BMP, and BMP/CS groups, we conducted flow cytometric analysis and immunofluorescence staining (Fig. 4a). We confirmed that there were 2.33% ± 1.26%, 3.85% ± 1.90% and 3.69% ± 1.73% CD45^−^Ter119^−^CD31 ^+^ Sca-1^+^ total endothelial cells (total ECs) in the PTs from the PBS, BMP, and BMP/CS groups, respectively (Fig. 4b, c).

Moreover, arteriolar blood vessels and type H vessels serve as functional blood vessel subtypes that can couple angiogenesis and osteogenesis.^16,17,37^ We found 2.62% ± 1.34% and 2.60% ± 1.04% CD45^−^Ter119^−^CD31^hi^Sca-1^hi^ arteriolar endothelial cells (aECs) in the PTs from the BMP and BMP/CS groups, respectively, which was much higher than that in the PBS group (0.33% ± 0.15%; Fig. 4b, d). The fractions of type H blood cells in the PTs from the BMP and BMP/CS groups were 2.66% ± 1.38% and 2.64% ± 1.05%, which were also much higher than that in the PBS group (0.35% ± 0.15%; Fig. 4b, e).

The immunofluorescence staining results also confirmed that the PTs from both the BMP and BMP/CS groups harbored abundant functional blood vessels, such as type H blood vessels (Fig. 4f, g). We further identified abundant Osterix^+^ osteoprogenitor cells intertwined with type H blood vessels in both the BMP and BMP/CS groups, which may indicate the vigorous osteogenic capacity of the newly generated PTs (Fig. 4h, i). Moreover, we found Aggrecan^+^ chondroprogenitor cells embedded in type H blood vessels in both the BMP and BMP/CS groups, which may indicate that the newly generated PTs were in an early transitional stage during the endochondral ossification process (Fig. 4j, k). No Osterix^+^ osteoprogenitor cells or Aggrecan^+^ chondroprogenitor cells were found in the PBS group. From the data above, we observed that newly generated PTs in the BMP and BMP/CS groups were abundant in functional blood vessels and skeletal progenitor cells and may possess a strong regenerative capacity in bone defect repair. The results confirmed the similarity between the newly generated PTs and natural periosteum. Although the quantitative data confirmed that no significant differences were observed in blood vessels between the two groups, the number of Osterix^+^ osteoprogenitors and Aggrecan^+^ chondrocytes in the BMP/CS group was significantly higher than that in the BMP group. These data indicated that the addition of CS could further increase the number of osteochondral progenitor cells in induced PTs and may enhance the osteogenic capacity of PTs.

### PTs accelerate calvarial reconstruction and enhance osseointegration via autologous transplantation

**Fig. 5:**
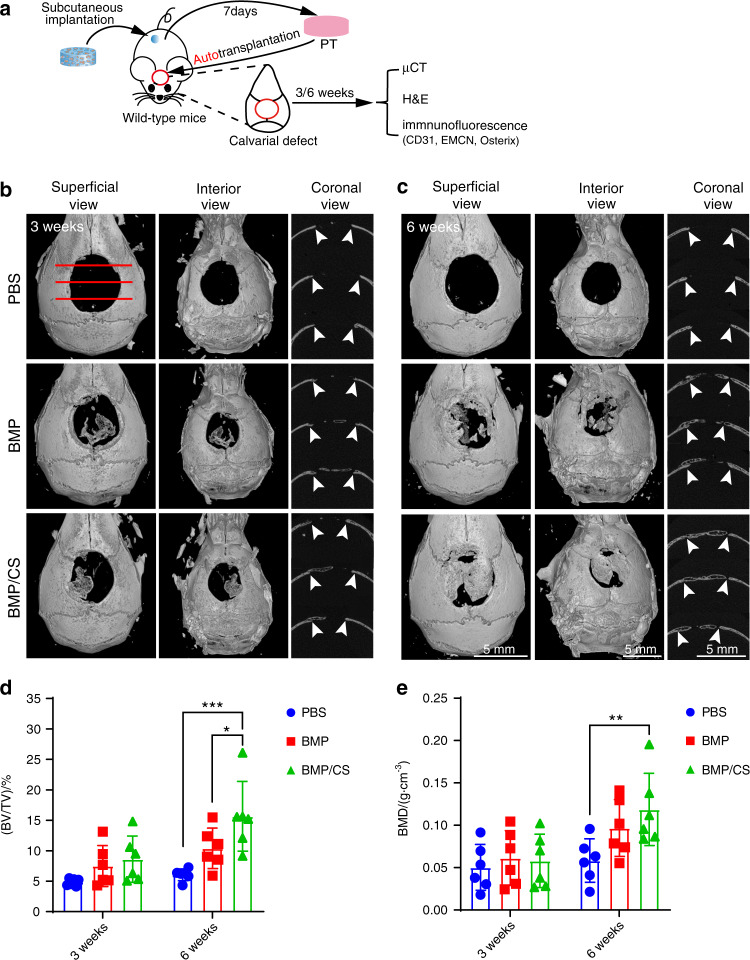
PTs accelerate calvarial reconstruction via autologous transplantation. **a** Scheme of autologous transplantation. PTs from the PBS, BMP, or BMP/CS groups were explanted and transplanted into the same mice with 5 mm diameter calvarial defects (*n* = 6). **b**, **c** Superficial, interior and coronal views of the cranium at weeks 3 (**b**) and 6 (**c**) after surgery. Images were taken at timed intervals for animals transplanted with PBS-, BMP-, or BMP/CS-induced autologous PTs. The white arrowhead indicates the integration site. Scale bar, 5 mm. **d**, **e** Quantitative analysis of bone volume fraction (BV/TV, **d**) and bone mineral density (BMD, **e**). Data were from individual animals and are shown as the mean ± SD.; *P < 0.05, **P < 0.01, two-way ANOVA followed by Bonferroni’s post hoc tests

To further evaluate the regenerative capacity of PTs, we conducted an autologous transplantation assay. In this study, we first fabricated a critical-sized calvarial defect model and transplanted autologous PTs into the defect area in the same mice (Fig. 5a). We observed only a few new bones in both the BMP and BMP/CS groups at week 3 after transplantation, whereas the amount of new bone dramatically increased at week 6 after transplantation (Fig. 5b–d). Specifically, there were no significant differences in the bone volume/total volume (BV/TV) ratio among all three groups at week 3 after transplantation, but the BV/TV ratio in the BMP/CS group was significantly higher than that in the BMP and control groups at week 6 after transplantation (Fig. 5b–d). The bone mineral density (BMD) in the BMP and BMP/CS groups showed a slight increase relative to the value in the PBS group at week 3 after transplantation, and the increase became even larger at week 6 after transplantation compared to the increase at week 3 after transplantation (Fig. 5e). However, the BMD value showed no significant difference between the BMP and BMP/CS groups (Fig. 5e). These data may indicate that the addition of CS could significantly increase the osteogenic volume and area of transplanted PTs during bone repair, but CS could only achieve a relative promotion of the mineralization of transplanted PTs.

**Fig. 6:**
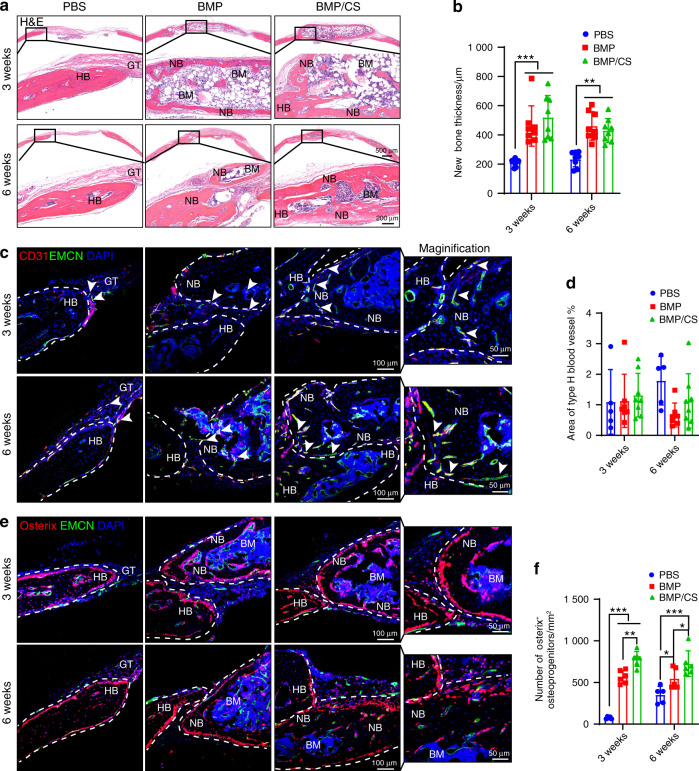
Induced PTs enhance osteogenesis and osseointegration during autologous transplantation for calvarial defect repair. **a**, **b** H&E staining (**a**) and quantitative analysis (**b**) were used to assess the integration of transplanted PTs with host bones (HBs) (*n* = 8). For PT in the PBS group, only new bone differentiated from HBs was observed. For PT in the BMP group, the mineralized HBs and the newly generated bones were partly separated by soft fibrous tissue. In contrast, HBs completely integrated with newly generated bones differentiated from the transplanted PTs in the BMP/CS group. **c**–**f** Immunofluorescence staining and quantitative analysis of type H blood vessels (EMCN ^+^ CD31 ^+^, yellow; **c**, **d**) and Osterix^+^ osteoprogenitors (red; **e**, **f**) in the PBS, BMP, and BMP/CS groups at weeks 3 and 6 after autogenic transplantation in young mice. Bone marrow BM, new bone NB, host bone HB, granulation tissue GT. EMCN-positive cells are green, CD31- and Osterix-positive cells are red, and DAPI is blue (nucleus). The white arrowhead indicates type H blood vessels (**c**). The white dashed line indicates the host bone or new bone area (**c**, **e**). Scale bar (top: low magnification, 1 mm; bottom: high magnification, 200 μm; **a**) and (left: low magnification, 100 μm; right: high magnification, 50 μm; **c**, **e**). Data are presented as the mean ± SD. *P < 0.05, **P < 0.01, ***P < 0.001, two-way ANOVA followed by Bonferroni’s post hoc tests

Histological analysis also confirmed the microcomputed tomography (μCT) imaging data, in which both the BMP and BMP/CS groups exhibited enhanced osteogenesis and osseointegration relative to the PBS group at weeks 3 and 6 (Fig. 6a, b) after transplantation. Immunofluorescence staining confirmed that PTs from the BMP and BMP/CS groups could facilitate the generation of vascularized new bone accompanied by abundant Osterix^+^ osteoprogenitors (Fig. 6c–f). Among all three groups, the BMP/CS group exhibited stronger osseointegration than the BMP group (Fig. 6c, e). Moreover, the number of Osterix^+^ osteoprogenitors in the BMP and BMP/CS groups was much higher than that in the PBS groups, and CS further increased the abundance of Osterix^+^ osteoprogenitors in newly generated bone (Fig. 6e, f). Considering that CS could not significantly increase the new bone thickness and type H blood vessel fraction in the BMP/CS group compared to the BMP group during bone repair, the increase in the osteogenic volume and area of transplanted PTs during bone repair may be due to the increase in osteoprogenitor cells in the BMP/CS group compared to the BMP group.

**Fig. 7:**
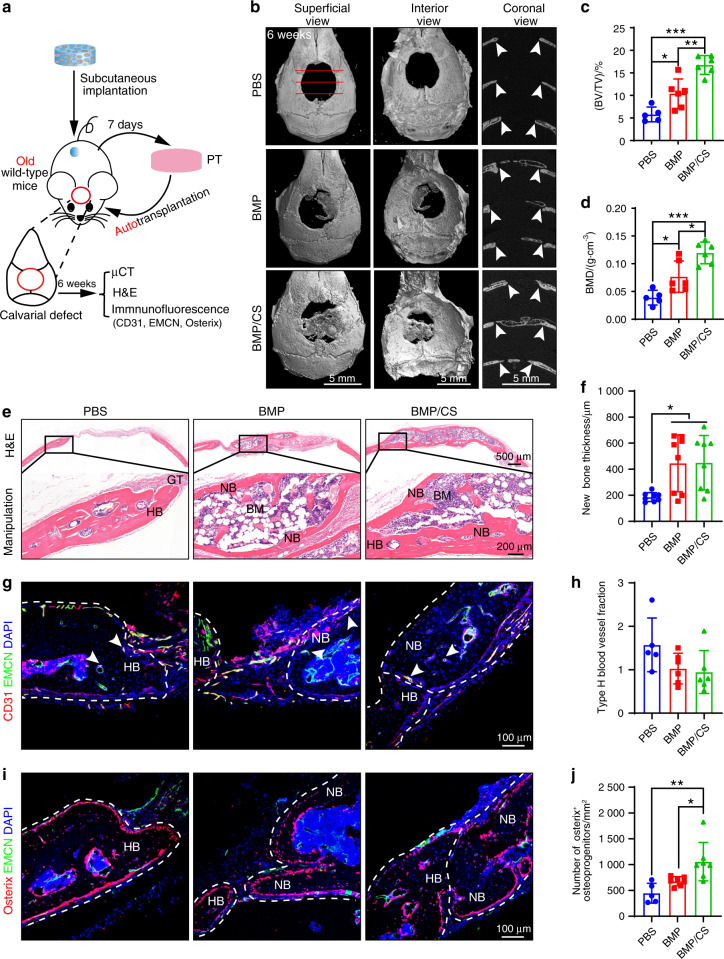
Induced PTs accelerate calvarial reconstruction via autologous transplantation in old mice. **a** Scheme of autologous transplantation in old wild-type mice. **b** Superficial, interior and coronal views of the calvaria at week 6 after surgery (*n* = 5–6). Images were taken at week 6 for animals transplanted with PBS-, BMP-, or BMP/CS-induced autologous PTs. The white arrowhead indicates the integration site. Scale bar, 5 mm. **c**, **d** Quantitative analysis of BV/TV (**c**) and BMD (**d**). **e**, **f** H&E staining (**e**) and quantitative analysis (**f**) were used to assess the integration of transplanted PT with host bone (HB) (*n* = 8). **g**–**j** Immunofluorescence staining and quantitative analysis of type H blood vessels (EMCN ^+^ CD31 ^+^, yellow; **g**, **h**) and Osterix^+^ osteoprogenitors (red; **i**, **j**) in the PBS, BMP, and BMP/CS groups at week 6 after allogenic transplantation in old mice (*n* = 5–7). Bone marrow BM, new bone NB, host bone HB, granulation tissue GT. EMCN-positive cells are green, CD31- and Osterix-positive cells are red, and DAPI is blue (nucleus). The white arrowhead indicates type H blood vessels (**g**). The white dashed line indicates the host bone or new bone area (**g**, **i**). Scale bar (top: low magnification, 500 μm; bottom: high magnification, 200 μm; **d**) and 100 μm (**f**, **h**). Data are presented as the mean ± SD. *P < 0.05, **P < 0.01, ***P < 0.001, one-way ANOVA followed by Tukey’s post hoc tests

Bone regenerative and reconstructive capacity declines with aging, and critical-sized bone defects have become a major clinical challenge in elderly individuals.^38–40^ Given this, we attempted to verify whether autologous PTs could still efficiently repair critical-sized bone defects in elderly individuals (Fig. 7a). μCT imaging confirmed that the BMP- and BMP/CS-induced PTs repaired much greater defect areas than those of the PBS group at week 6 after transplantation (Fig. 7b). We further confirmed that the BV/TV ratio and the BMD were higher in both the BMP group and the BMP/CS group than in the PBS group (Fig. 7c, d). Among all three groups, the BMP/CS group had the highest value for both BV/TV and BMD (Fig. 7c, d).

Histological analysis also confirmed that the PTs in the BMP and BMP/CS groups generated much more new bone than the PTs in the PBS group (Fig. 7e, f). Moreover, the newly generated bones differentiated from the PTs in the BMP/CS group could completely integrate with host bones (HBs), whereas the mineralized HBs and the newly generated bones were partly separated by soft fibrous tissues in the BMP group. The newly generated bones in the PBS group were all derived from mineralized HBs. Immunofluorescence staining also confirmed that the newly generated bones were highly vascularized and abundant in osteoprogenitor cells (Fig. 7g–j). All these data confirmed that newly generated PTs could repair critical-sized bone defects via autologous transplantation in both young and old mice and that CS could further enhance this reconstructive capacity.

## Discussion

The periosteum is a fibrous-like tissue enriched in abundant functional blood vessels and skeletal stem cells that surrounds almost the entire bone surface and exhibits a strong regenerative capacity in bone defect repair.^10,12^ Constructing artificial PTs, which possess anatomical structures similar to those of the natural periosteum and harbor abundant functional blood vessels and PDCs, has become a promising strategy for critical-sized bone defect repair, especially for elderly patients.^20,21,31^ Previous periosteum-mimetic strategies mainly focus on mimicking the anatomical structure and cellular component of the periosteum via multiple synthetic or natural materials and exogenous cells, which ignore the direct and effective activation of the residual regenerative capacity of individuals in vivo.^41–46^ In this study, we provided an in vivo approach for creating PTs by subcutaneously implanting BMP-2-loaded scaffolds that mimicked mesenchymal condensation during endochondral ossification. We confirmed that BMP-2 was indispensable for fabricating PTs and that the PTDCs lodged in the PTs were deeply involved in the reconstruction of allogenic critical-sized calvarial defects. Furthermore, we confirmed that the synergy of BMP-2 and biological materials, such as CS, could regulate the function of PTDCs in PTs and may contribute to the enhanced osteogenesis and osseointegration of PTs in subsequent autologous transplantation assays in both young and old mice.

In this study, we chose absorbable gelatin sponges as the carrier materials for BMP-2 since absorbable gelatin sponges are a widely clinically used (FDA approved) and cost-effective product. The combination (FDA-approved carrier materials and BMP-2) adopted in this strategy would facilitate clinical translation compared to other synthetic and natural materials. Moreover, absorbable gelatin sponges have been used clinically for decades, and little doubt regarding their biocompatibility and immunogenicity has been reported.^47–50^ The key points for this strategy are the chosen bioactive proteins, such as BMP-2 and BMP-7, and the timing for harvesting PTs, which varies with the dose of proteins loaded in biomaterials. This cell-free strategy can utilize many kinds of bioactive protein and biomaterial combinations as long as these combinations could trigger endochondral ossification.^51,52^

Extensive vascularization is an important feature of the periosteum.^3,8,10^ Functional blood vessels strongly affect the osteogenesis and integration of bone transplant in bone defect areas.^16,17^ Abundant functional CD31^hi^Emcn^hi^ type H vessels emerged in the PTs in both the BMP and BMP/CS groups, which was similar to the vascularization status in the natural periosteum. Previous studies have proven that TRAP ^+ ^macrophage-lineage cells can secrete PDGF-BB to induce the formation of CD31^hi^Emcn^hi^ type H vessels and recruit PDCs, such as LepR^+^ and CD105 ^+ ^PDGFRa^+^ PDCs.^10,14,15,53^ Interestingly, we found many TRAP ^+ ^macrophage-lineage cells in the PTs from the BMP and BMP/CS groups, and these cells may contribute to the formation of highly vascularized and osteochondral progenitor cell-abundant PTs. We indeed confirmed that the PTs harbored an extremely high fraction of type H blood vessels and arteriolar blood vessels. These vascularized and osteochondral progenitor cell-abundant PTs may be further applied to bone defect repair. In addition to being highly vascularized, the periosteum is also abundantly innervated.^54,55^ Abundant periostin emerged in the PTs, indicating that the generation of PTs may be regulated by nerves to some extent.

Previous studies have confirmed that CS could stabilize BMP-2 loaded in implanted scaffolds.^36,56,57^ In this study, we confirmed that CS, as an essential component in the extracellular matrix (ECM), could further enhance the osteogenesis and chondrogenesis of PTDCs recruited from PTs. This enhanced differential capacity of PTDCs may be due to the prolonged half-life of BMP-2. Moreover, the negative charge of CS facilitated binding to cations, such as calcium ions, which promoted the osteogenesis of recruited PDCs.^35^ Typically, PDCs home to the injury site via a classic CXCL12/CXCR4 axis for tissue regeneration.^58–61^ Here, we unexpectedly found that CS increased the expression level of *CXCL12* in PTDCs from the PTs in the BMP/CS group, which in turn recruited more regenerative-associated progenitor cells. This phenomenon may be the reason why many more PTDCs were lodged in the PTs from the BMP/CS group than in the PTs from the BMP group. The high expression of *SCF* in the PTDCs from the PTs in the BMP/CS group would also facilitate maintenance of the quiescent state of recruited hematopoietic stem cells and accelerate the formation of the marrow cavity in newly generated bone tissue. Allogenic transplantation assays also confirmed that the LepR^+^ PTDCs from the PTs in the BMP/CS group exhibited an enhanced osteogenic capacity compared to the PTDCs from the PTs in the BMP group.

Subsequent autologous transplantation assays showed that the PTs in both the BMP and BMP/CS groups could enhance osteogenesis and facilitate calvarial defect repair. CS served as an enhancer and further improved the osteogenesis and osseointegration of the transplanted PTs in the BMP/CS group compared to the transplanted PTs in the BMP group, even in old mice. In old individuals, the senescence of PDCs and the decreased function of blood vessels hindered the regenerative capacity.^62,63^ It is preferable to fabricate highly vascularized and osteochondral progenitor cell-abundant PTs in undamaged and blood supply-rich sites since the blood supply and the abundance of osteochondral progenitor cells are commonly insufficient in defect sites in old individuals. Moreover, the optimal incubation time and dosage of BMP and CS for fabricating PTs require further analysis for confirmation.

This work presents evidence of a novel approach to create PTs with the aid of BMP-2-loaded scaffolds. PTs were abundant in PTDCs and functional blood vessels. Moreover, the bone regenerative capacity of the PTs could be regulated by directly controlling the properties of biomaterials. We found that CS, which served as an enhancer, could synergistically enhance the osteogenesis and osseointegration of PTs by increasing the abundance and differential capacity of the recruited PTDCs. Although the specific cellular mechanism for the enhanced regenerative capacity of the PTs arising from CS still needs further investigation and more types of enhancers should be identified, this strategy provides an effective platform for screening vital cellular mechanisms and functional properties of the natural periosteum by varied material combinations. Other negatively charged glycosaminoglycans, such as KS (keratan sulfate), DS (dermatan sulfate) and hyaluronan (HA), may possess the capacity to stabilize BMP-2 and bind Ca^2+^ ions and deserve further investigation in our following study. Lineage-tracing assays for dissecting the origin of PTDCs in PTs would be a key point in our following investigation. As the periosteum plays a central role in bone defect healing and modeling, these developmentally inspired PTs derived from an individual and lacking immunological rejection deserve further clinical translation.

## Materials and methods

### Fabrication and characterization of scaffolds

We fabricated PBS-, BMP-, and BMP/CS-loaded gelatin scaffolds with a conventional freeze-drying method. Briefly, we added recombinant human BMP-2 (rhBMP-2) solution (30 μg rhBMP-2 in 60 μL of solution) or rhBMP-2 and CS mixed solution (30 μg rhBMP-2 and 30 μg CS each in 60 μL of solution) or phosphate buffer solution (PBS, 60 μL) onto a porous absorbable gelatin scaffold (5 mm in diameter and 5 mm in thickness, Jiangxi Xiangen Co., Ltd., China) under sterile conditions. Next, the PBS-, BMP-, and BMP/CS-loaded gelatin scaffolds were freeze-dried and stored at −20 °C for use. RhBMP-2 was provided by Shanghai Rebone Biomaterials Co., Ltd. CS was purchased from Shanghai Yuanye Biotechnology Co., Ltd. The mesoporous structures of the scaffold were characterized by field emission scanning electron microscopy (FE-SEM, Hitachi S-4800, Japan).

### Animals and animal assays

Wild-type C57BL/6 male mice aged 8–10 weeks and 52–56 weeks used in this study were purchased from the Experimental Animal Center of East China Normal University. *Lepr*-Cre; tdTomato mice were maintained on a C57BL/6 background and were kindly gifted from Professor Rui Yue from Tongji University.^14^ All mice were housed in the animal facility of East China Normal University. All of the experimental procedures were approved by the East China Normal University Animal Care and Use Committees.

We first anaesthetized the mice with 1% (w/v) pentobarbital sodium solution, shaved the hair on the back, and subcutaneously implanted PBS-, BMP-, and BMP/CS-loaded gelatin scaffolds. We then explanted the tissues induced in the PBS, BMP, and BMP/CS groups for further analysis or transplantation 1 week after implantation.

Specifically, for verification of the anatomical structure of the induced tissues and further evaluation of the function of the induced tissues in the PBS, BMP, and BMP/CS groups, *Lepr*-Cre; tdTomato mice were subcutaneously implanted with the scaffolds mentioned above. *Lepr*-Cre; tdTomato male mice were randomly divided into different groups (*n* = 6), and each mouse in the same group was subcutaneously implanted with two scaffolds.

To evaluate the function of PTDCs derived from PTs in the PBS, BMP and BMP/CS groups, we conducted allogenic transplantation in a critical-sized calvarial defect repair assay. We first fabricated PTs with PBS-, BMP- and BMP/CS-loaded scaffolds after 1 week of subcutaneous implantation in the *Lepr*-Cre; tdTomato mice. We then anaesthetized the mice and further cleared the hair on the back and head with the aid of depilatory cream. After the preparatory procedure, we separately explanted the PTs induced by the PBS-, BMP-, and BMP/CS-loaded scaffolds and sutured the wound. The PTs were then subjected to PBS and engraved with a thin-walled circular skin sampler (5 mm diameter). We then incised the skin-covered cranium and drilled a 5 mm diametric circular defect on the cranium with a cranial drill (5 mm external diameter) to construct critical-sized calvarial defects in wild-type mice. Finally, we placed the engraved PTs onto the defect area and sutured the skin in the wild-type mice. The mice were kept warm until they revived. The mouse craniums in the PBS, BMP and BMP/CS groups were collected for further study 3 or 6 weeks after implantation.

To evaluate the regenerative capacity of the PTs, we conducted autologous transplantation in a critical-sized calvarial defect repair assay. Wild-type male mice aged 8–10 weeks and 52–56 weeks were randomly divided into the PBS, BMP, and BMP/CS groups (*n* = 5–6 for transplantation assays in normal mice and *n* = 5–6 for transplantation assay in old mice), and each mouse in the same group was subcutaneously implanted with one scaffold. We first fabricated PTs with PBS-, BMP-, and BMP/CS-loaded gelatin scaffolds 1 week after subcutaneous implantation and transplanted the PTs to the defect area in the same wild-type mice. The mice were kept warm until they revived. The mouse craniums in all groups were collected for further study at the indicated times after implantation.

### Histochemical staining

Freshly explanted samples were fixed in 4% paraformaldehyde (PFA) overnight and decalcified in 0.5 mol·L^−1^ EDTA solution for another 3 days. The samples were embedded in paraffin and sectioned into 5 μm slices. The slices were then stained with hematoxylin/eosin (H&E), Safranin O/Fast Green, and TRAP using a standard protocol according to the manufacturer’s instructions. All mounted slices were imaged via digital slide scanners (Pannoramic MIDI) and analyzed with Fiji.

### Antibodies and staining reagents

For flow cytometry, BV480-anti-CD105 (MJ7-18) and BV650-anti-CD31 (390) from BD Bioscience; APC-CD140a (APA5), PE-Cy5-anti-Ter119 (Ter119), PE-Cy5-anti-CD45 (30-F11), and AF700-anti-Ly6A/E (D7), all from eBioscience; FITC-anti-Ter119 (Ter119), FITC-anti-CD45 (30-F11), FITC-anti-CD31 (390), from Biolegend; and FITC-anti-Endomucin (EMCN) from Santa Cruz were used.

For immunofluorescence staining, the following primary antibodies were used: rabbit anti-mouse CD31, rabbit anti-mouse Aggrecan, rabbit anti-mouse Osterix, and rabbit anti-mouse periostin all from Abcam; and rat anti-mouse EMCN from Santa Cruz; the secondary antibodies were as follows: goat-anti-rabbit-Alexa Fluor 555, goat-anti-rat-Alexa Fluor 488, and goat-anti-rabbit-Alexa Fluor 488, all from Abcam. Prolong gold antifade reagent with DAPI from CST was used.

### Flow cytometry

For endothelial cell and PTDC staining, the residual scaffolds in PTs were first discarded, and then, the freshly obtained PTs were crushed in staining buffer (Biolegend) with a mortar and pestle. Single-cell suspensions were obtained with an additional digestion in collagenase solution (Roche Diagnostics) at 37 °C for 30 min.

Cell suspensions were then filtered with a 40 μm cell strainer and spun at 300 × *g* for 5 min at 4 °C. The supernatant was removed, and the deposit was resuspended in staining buffer. Then, the resuspended single cells were stained with an antibody cocktail for 60 min at 4 °C. After incubation, the cells were washed and resuspended in adequate staining buffer again and used for flow cytometric analysis.

Flow cytometric analyses were carried out using a CytoFlex LX system equipped with CytExpert 2.3 software (Beckman Coulter) or a Symphony A5 system equipped with Diva 8.0 software (BD Biosciences). Dead cells were excluded using a Live/Dead Fixable Near-IR Dead Cell Stain Kit (Thermo Fisher Scientific) or DAPI solution (1 μg·mL^−1^). We used fluorescence minus one control to define the boundaries between the positively and negatively stained cell populations. Flow cytometric data were analyzed with FlowJo V10 (Three Star) or CytExpert 2.3.

### Immunofluorescence staining

For immunofluorescence staining, decalcified samples were immersed in 20% sucrose solution overnight, embedded in optimum cutting temperature compound and sectioned with a cryotome cryostat (at −20 °C) to 10 µm or 40 µm thickness. The sections were left at room temperature (RT) for 15 min and then immersed twice in pure water for 5 min each time. The sections were then removed and rinsed three times with PBST (1 mL of Tween 20 in 1 L of 0.01 mol·L^−1^ PBS) and blocked with 10% (v/v) goat serum at RT for 1 h. After blocking, the sections were stained with CD31 (1:100), Osterix (1:200), Aggrecan (1:400), EMCN (1:100), and periostin (1:200) overnight at 4 °C. As appropriate, the sections were stained with secondary antibodies labeled with Alexa Fluor 488 (1:400) and Alexa Fluor 555 (1:400) at RT for 1 h and mounted with Prolong gold antifade reagent with DAPI (CST). The mounted sections were imaged via laser scanning confocal microscopy (Leica SP8) and analyzed with Fiji.

### CFU-F assay

We first cultured PTDCs from the explanted PTs induced by BMP and BMP/CS-loaded gelatin scaffolds as described in the protocol.^13,14^ Briefly, we digested the PTs to obtain a single-cell suspension. For the CFU-F assays, we plated the obtained single-cell suspensions at a density of 1 × 10^4^ cells/well in 6-well plates supplied with α-MEM (Gibco) plus 20% fetal bovine serum (Gibco), 10 μmol·L^−1^ ROCK inhibitor (Y-27632, MCE), and 1% penicillin/streptomycin (Invitrogen). The cultures were maintained at 37 °C in a humidified, gas-tight chamber (Thermo Fisher) containing 5% O_2_ and 5% CO_2_ to enhance progenitor survival and proliferation. CFU-F colonies were counted after 8 days of culture by staining with 0.1% toluidine blue in 4% PFA solution.

### qPCR of PTDCs

We first cultured the digested single-cell suspension mentioned above at a density of ~1 × 10^7^ cells/100-mm dishes in α-MEM (Gibco) with 20% FBS qualified (Gibco), 10 μmol·L^−1^ ROCK inhibitor (Y-27632, MCE) and 1% penicillin/streptomycin (Invitrogen). We washed the adherent cells with PBS and replaced the medium with 15 mL of fresh complete medium after an additional 12 h of culture. We changed the culture medium every 3 days. When the cell confluence reached 80%–90%, we digested adherent cells and extracted total RNA from the digested PTDCs using TRIzol reagent (TaKaRa) according to the manufacturer’s protocol. Reverse transcription of mRNA was performed using a PrimeScript RT reagent kit (TaKaRa) as recommended by the manufacturer’s protocol. qPCR was performed using a Bio-Rad 9600 machine (Bio-Rad) with SYBR Green PCR Master Mix (TaKaRa). *Gapdh* was used to normalize the RNA content of samples. The primer sequences used were as follows: *Gapdh*: 5′-AAATGGTGA AGGTCGGTGTGAAC-3′ and 5′-CAACAATCTCCACTTTGCCACTG-3′; *Sox9*: 5′-GAGCCGGATCTGAAGAGGGA-3′ and 5′-GCTTGACGTGTGGCTTGT TC-3′; *Acan*: 5′-CCTGCTACTTCATCGACCCC-3′ and 5′-AGATGCTGTTGACTCGAAC CT-3′; *Runx2*: 5′-TTACCTACACCCCGCCAGTC-3′ and 5′-TGCTGGTCTGGAAGGGT CC-3′; *OPN*: 5′-AGCAAGAAACTCTTCCAAGCAA-3′ and 5′-GTGAGATTCGTCAGA TTCATCCG-3′; *CXCL12*: 5′-TGCATCAGTGACGGTAAACCA-3′ and 5′-TTCTTCA GCCGTGCAACAATC-3′; *SCF*: 5′-GCCAGAAACTAGATCCTTTACTCCTGA-3′ and 5′-CATAAATGGTTTTGTGACACTGACTCTG-3′.

### In vivo imaging

To evaluate the survival and proliferation of PTDCs from transplanted PTs in the PBS, BMP, and BMP/CS groups after allogenic transplantation, we conducted an in vivo imaging assay. The mice treated with PTs from the PBS, BMP, and BMP/CS groups were imaged at week 6 after transplantation using IVIS Spectrum CT (Perkin Elmer). The mice were imaged as a standard mouse model for fluorescence and CT imaging. The resolution for CT was 425 μm. Fluorescence intensity was analyzed by Living Image Software 4.4.

### μCT imaging

For the cranium collected from autologous transplantation assays, we used high-resolution µCT (Skyscan 1272, Skyscan) to evaluate the repair effect. The scanner was set at a voltage of 60 kV, a current of 160 µA and a resolution of 9.0 µm per pixel. BV/TV and BMD were calculated with CTAn.

### Statistical analysis

The statistical analysis was conducted using unpaired, two-tailed Student’s *t* tests between two groups. For more than two groups, the statistical analysis was conducted with one-way ANOVA, followed by Tukey’s multiple comparison tests, or two-way ANOVA, followed by Bonferroni’s multiple comparison tests, as indicated in the figure caption. All data represented as the mean ± SD were analyzed with GraphPad Prism 7. *P < 0.05, **P < 0.01, and ***P < 0.001.

## Supplementary information

Supplementary Figs. 1-3
