# Supplementary material for: Construction of developmentally inspired periosteum-like tissue for bone regeneration
Source: Bone Res. 2022 Jan 3;10:1. doi: 10.1038/s41413-021-00166-w (PMC8720863; doi:10.1038/s41413-021-00166-w)
Supplement: Supplementary file 1 — Supplementary Figs. 1-3 [file 41413_2021_166_MOESM1_ESM.docx]

# Supplemental Figures and Figure Legends

# Construction of developmentally inspired periosteum-like tissue for bone regeneration

Kai Dai^a,c^, Shunshu Deng^a,c^, Yuanman Yu^a,c^, Fuwei Zhu^a,c^, Jing Wang^a,c,*^& Changsheng Liu^b,c,d*^

^a^State Key Laboratory of Bioreactor Engineering, East China University of Science and Technology, Shanghai 200237, PR China

^b^Key Laboratory for Ultrafine Materials of Ministry of Education, East China University of Science and Technology, Shanghai 200237, PR China

^c^Engineering Research Center for Biomedical Materials of Ministry of Education, East China University of Science and Technology, Shanghai 200237, PR China

^d^Frontiers Science Center for Materiobiology and Dynamic Chemistry, East China University of Science and Technology, Shanghai, 200237, PR China


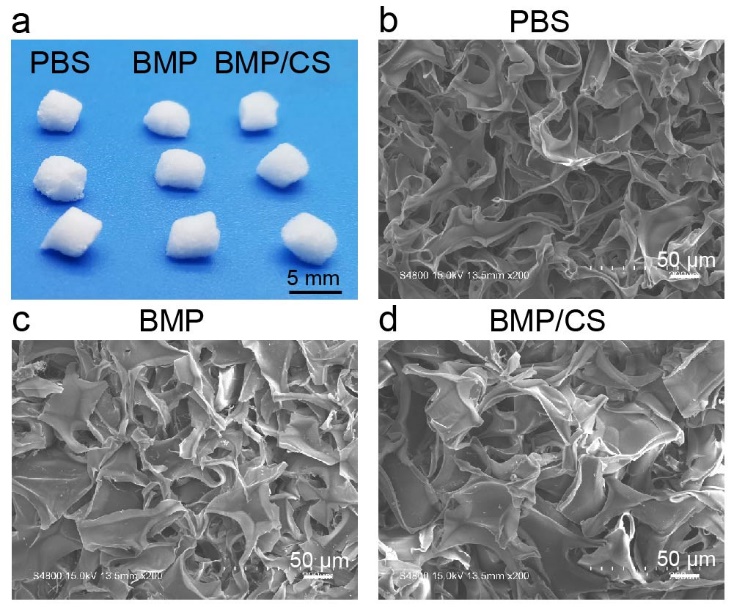


**Supplementary Fig. 1. Gross appearance and SEM images of gelatine sponges.** **a** Representative gross images of gelatine sponges treated with PBS, BMP-2, and BMP/CS. **b-d** Representative images of SEM scanning of gelatine sponges. Scale bar, 50 μm.


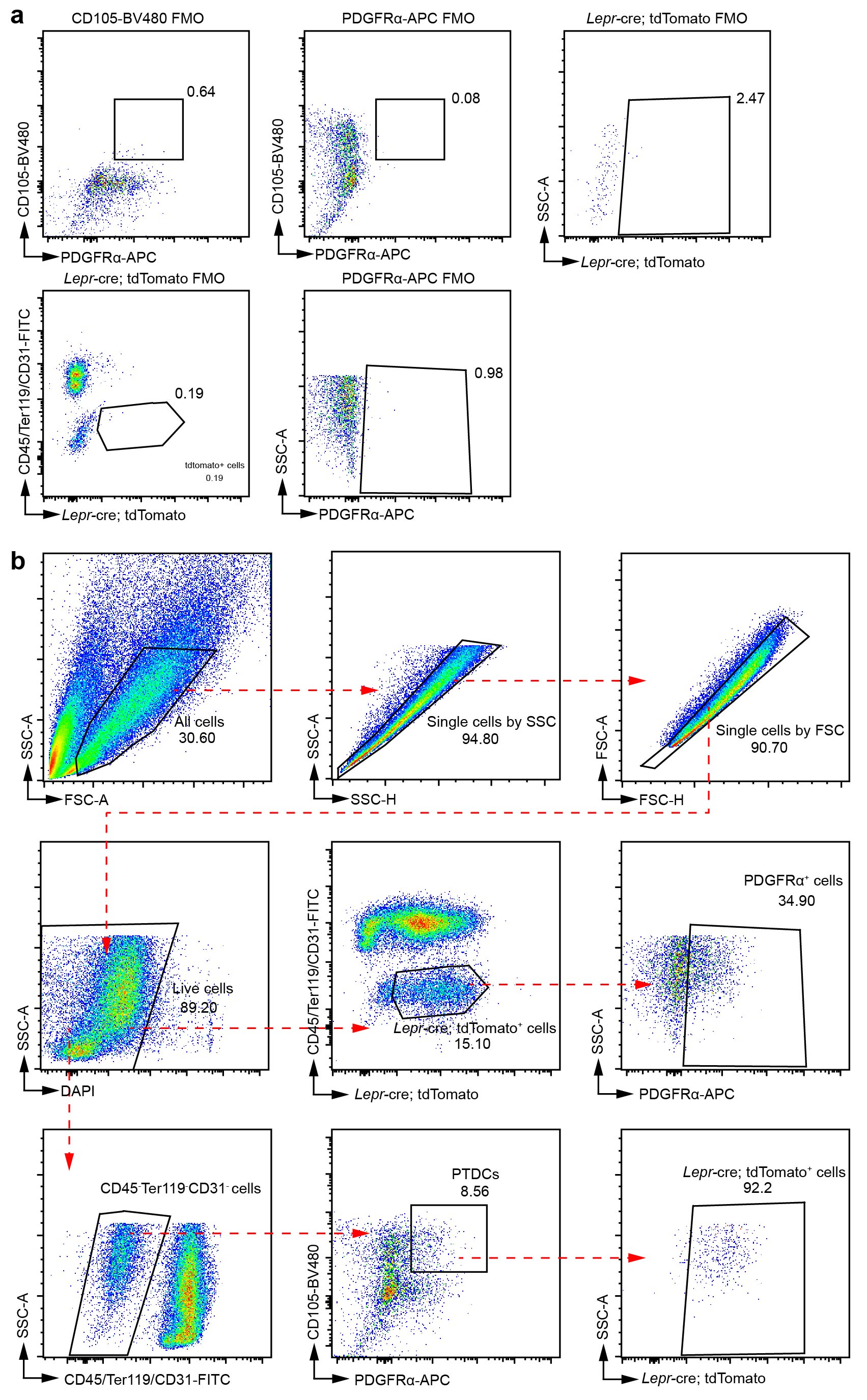


**Supplementary Fig. 2. Fluorescence-minus-one (FMO) controls and gating strategy for PTDCs from induced PTs.** **a** Representative flow cytometric plots for each FMO control (e.g., anti CD105 in BV480, anti PDGFRα in APC, LepR in tdTomato) are shown. **b** Gating strategy for LepR^+^ progenitor cells and PTDCs from induced PTs. Representative flow cytometric plots with the percentage of parent gate are shown for each population.


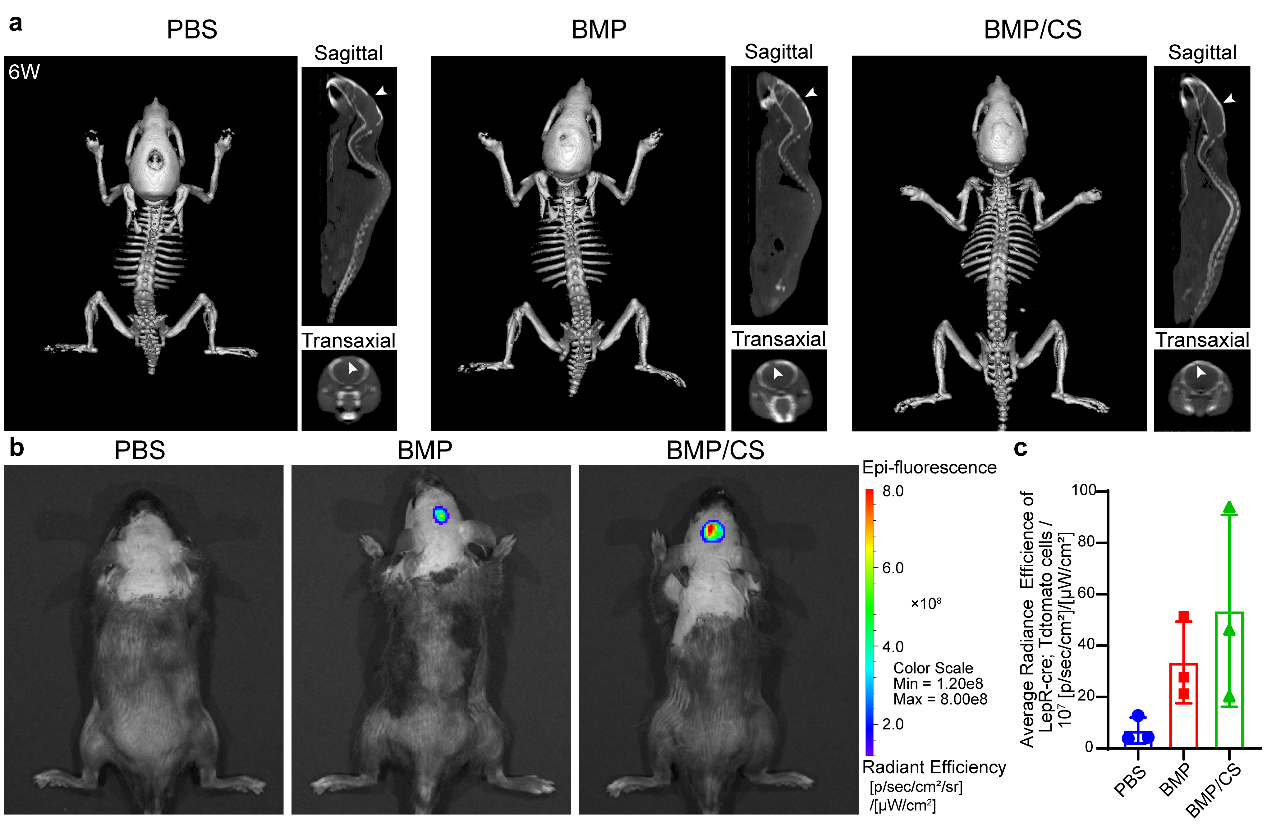


**Supplementary Fig. 3. *In* vivo imaging of PTDCs from transplanted PTs at week 6 after allogenic transplantation.** **a** Representative CT images are shown as vertical view, sagittal view, and transaxial view for each mouse. The white arrowhead indicates the defect area. **b-c** Representative fluorescence images (b) and quantitative analysis (c) are shown (n = 3). Data represented as the mean ± SD. One-way ANOVA, followed by Tukey’s multiple comparison tests.
